# Examining the dynamic impact of carbon emissions, renewable energy and economic growth on healthcare expenditure in Asian countries

**DOI:** 10.1016/j.heliyon.2024.e30136

**Published:** 2024-04-23

**Authors:** Preeti Slathia, Ashutosh Vashishtha, Pabitra Kumar Jena, Pritish Kumar Sahu

**Affiliations:** a School of Business, Shri Mata Vaishno Devi University, Kakryal, 182320, Jammu & Kashmir, India; b School of Economics, Shri Mata Vaishno Devi University, Kakryal, 182320, Jammu & Kashmir, India; c International Management Institute- IMI, Gothapatna, Chandaka Malipada, Bhubaneswar, Odisha, India, 751003; d Central University of Jammu, Rahya Suchani, Samba, 181143, Jammu & Kashmir, India

**Keywords:** CO_2_ emissions, Renewable energy, Economic growth, Health expenditure, Asian economies, FMOLS and DOLS

## Abstract

This study aims to examine the impact of renewable energy, carbon emissions, and economic growth on healthcare spending in 36 Asian countries during 2000–2019. Fully Modified Ordinary Least Square (FMOLS) and Dynamic Ordinary Least Square (DOLS) models have been applied to the panel data for 36 Asian countries. The study's findings show that CO_2_ emissions in Asia increased due to public and private health spending, with the commercial health sector having a larger negative influence on CO_2_ emissions than the public sector. According to FMOLS and DOLS findings, carbon emissions and GDP are positively related to health spending, indicating that high economic growth through energy-intensive production processes leads to increased carbon emissions, but on the contrary, renewable energy consumption has decreased healthcare expenditure. This study advocates new policies to reduce carbon emissions and hospitalisation without jeopardising national economic growth. In order to achieve sustainable health services and an environmentally friendly future in Asia, health administrators must raise state and private healthcare spending while implementing an effective cost-service and energy-efficient management plan.

## 1 Introduction

The ability of ordinary people to freely ventilate in their surroundings is the century's main challenge. The continuous increase in greenhouse gases, primarily carbon dioxide, dealt a severe blow to the ecosystem and etched a threat to humanity's survival*. According to* Ref. [1,2]*, carbon dioxide is a well-known greenhouse gas emitted by human activities that contribute to global warming and have negative effects on human health*. Many industrialized and developing nations sacrifice environmental health and air quality in the name of quick production growth. The environment's degradation and the deterioration of the atmosphere make spending money on medical treatments more necessary to maintain a healthy lifestyle (Alimi et al., 2019). The exhaustive usage of natural resources to broaden the manufacturing process. The remarkable growth in the field of economic and social development leads to the deterioration of natural resources. *The scarcity of energy sources and constant aggressive consumption made government houses around the globe maintain sustainable policies for the environment*. Environmental deterioration, intensive energy use, economic growth, and human health are terrifying threats to human welfare and health [3]. The cost of health care spending is impacted by the rise in environmental pollution brought on by human emissions such as CO_2_ (Ahmad et al., 2021 and Alhassan, H., & Kwakwa, P. A [4]. Public funding for better health care facilities and affordable health protection is the reason behind the constant rise of healthcare expenditures. The development of cities, industrial growth, rising usage of energy, construction of infrastructure, and migration from the countryside to the cities are all contributing factors to the increasing need for health care facilities and insurance (Apergis et al., 2020). Increased spending on healthcare are an indicator of a couple of factors: (a) societal sensitivity to the need to maintain personal and community wellness; and (b) a substantial amount of manufacturing output, which can lead to airborne exposure to chemicals and malnutrition, which can cause malignancy (Gerdtham and Jönsson 2000). However, the increased level of pollution lured the researchers to explore the relationship between economic growth, energy consumption, and environmental degradation [5,6], enormous research has already been conducted to make reductions in greenhouse gases, particularly carbon dioxide. So, it becomes even more important to analyze how the environment, renewable energy consumption, health care spending, and economic growth interact (see Table 15, Table 16).

It is estimated that about 70 % of energy consumption is met by non-renewable energy (“World Development Indicator” 2018) in Asian Economies. While multiple commitments have been made about how to combat climate change by authorities, legislatures, scientists, and governments, the most vital objective is for economies to migrate from energy generated from fossil fuels to alternative sources of energy (Avagyan 2021, Kwakwa, P. A. [7], & Gyamfi, 2023). The paradigm shift shows the conservation of energy and mitigation of carbon emissions and other greenhouse gases. Over the decades, greenhouse gases have escalated abruptly, creatingrightening situations such as global warming and climate change [8]. Different Asian nations made a variety of initiatives such as investing and promoting cleaner transportation, energy-efficient housing, power generation, industry, and better municipal waste management to address the severe challenges posed by exhaustive usage of resources, intensive energy use, growth in the economy.

The relationship between health spending, economic growth, and energy consumption has garnered greater consideration in developed and developing countries in recent decades. Four categories emerged from the available research on energy consumption, health care costs, and economic growth. The first set of research examined the connection between environmental sustainability and economic growth. The Environmental Kuznets Curve (EKC) framework, which demonstrated an inverse U-shaped link between environmental damage and growth in the economy, was employed in these investigations. First proposed by Grossman and Krueger in 1995, EKC contends that as wealth rises, environmental quality begins to deteriorate, reaches a point of saturation, and progressively diminishes (Inglesi-Lotz and Dogan, 2018). The second body of literature addressed healthcare spending and growth in the economy. The distribution of healthcare expenses (Fan and Savedoff 2014; Dieleman et al., 2016) and the financial flexibility of medical expenses (Bustamante and Shimoga 2018) were among the topics covered in the aforementioned research. Yu et al. (2016) conducted an analysis of the relationships between healthcare spending and CO_2_ emissions in the third category of literature. On the other hand, few studies looked at the relationship between CO2 emissions and health care spending, specifically examining how CO_2_ emissions are impacted by health care spending and energy consumption. Finally, the fourth strands the interlink between renewable energy and environment degradation. Globally, renewable energy consumption continues to trend higher, but CO_2_ emissions have also risen, increasing from 20 million kt in 1990 to 34 million kt in 2019 (World Bank, 2023a). Numerous studies using empirical methods have demonstrated that the consumption of renewable energy lowers the level of carbon dioxide pollution (Aydin et al., 2023; Kwakwa, 2021. [9], 2022; Piłatowska et al., 2020; Ridzuan et al., 2020). While green energy has an opportunity to lower carbon emissions, certain investigators have discovered that green energy raises the amount of carbon dioxide emission (Avagyan and Singh, 2019; Yang et al., 2022; Pata and Kartal, 2023). Many scientific studies have utilized statistics from America, Europe, and Asia (Murshed et al., 2022; Hasnisah et al., 2019).

In the given scenario, it is certitude that carbon emission is the overriding cause of climate change, studies by Ref. [[10], [11], [12], [13], [14]] support the fact [15]. who identified air pollution (carbon dioxide) as the primary cause of ozone, with increased UV radiation hurting human health, death, and hospitalisation in his study. The rising death toll and healthcare costs impact manufacturing processes and economic growth. Carbon Emissions in China causes a human health crisis, environmental degradation, and economic growth. Zhang et al. [16].

It is noteworthy that, with the exception of China, emissions from emerging market and developing economies in Asia increased by 4.2 % or 206 Mt CO_2_ in 2022, outpacing emissions from all other regions. The region's emissions increased by more than half as a result of coal-fired power plants International Energy Agency [17], indicating the role of Asia's greenhouse gas effect is much higher than in any other country due to the cutting-edge rise in population, increased rate of energy consumption, scarcity of energy resources, and meagre management of resources. China heavily contributed to the total increase in Asia subregion environmental impact Galli et al. [18]. However, West and Central Asia also show a high rise in per capita growth compared to other regions. The region's increased per capita environmental impact grew by 126 % and 146 % between 1960 and 2016 and the population growth was 155 % and 465 % respectively Lin et al. [19]. The transformation in the demographic structure, and energy levels due to manufacturing processes and industries spreading out huge amounts of greenhouse gases, especially carbon emissions leading to more harmful effects in south Asia [18,20]. There is quite extinguishing growth shown in the case of Bhutan being the smallest region.

Since the 2000s, China has left behind the US over increased carbon emissions and huge economic growth for over 15 years. At present, China exceeds the list by 29.18 % world share, followed by the US by 14.02 %, and India by 7.09 %. In the present scenario, China's population shows a negative and steady trend due to imposing government policies and regulations. In the recent study from the (World Wildlife Fund (WWF) Living Planet Report [21], it was noted that both population and the exploitation of natural resources had caused environmental deterioration in Singapore.

Prior research focused less on the connection between renewable energy consumption and health, and even lesser on the correlation between health spending, economic growth and environment (CO_2_ emissions). The current research examines statistics from 36 Asian countries in an attempt to close this disparity. Due to their inadequate health care systems, many Asian nations are more susceptible to increasing healthcare stresses. Furthermore, it is a stimulating topic to look into considering the continent of Asia's tremendous population, brittle structures and lack of legislative appetite to invest in social assistance initiatives. The ecology is also greatly impacted by the patterns of energy usage in Asian economies (Murshed et al. b).

This study is novel from these prospects: Firstly, the present study employed to investigate the effect of carbon emissions, Renewable Energy and Economic Growth on Healthcare Expenditure, considering the environmental quality in 36 Asian countries, further grouping them into sub-groups Central Asia, East Asia, South Asia, South-East Asia and West Asia. Secondly, the research considers the undeviating impact of carbon emissions and Health expenditure and the incidental impact of Renewable Energy and Health expenditure. In contrast to prior research, our study utilized Health expenditure as Dependent Variable, employing FMOLS and DOLS to state the relationship between Renewable energy, Economic Growth and carbon emissions. In addition, coherent results are based on the theoretical foundation (IPAT model).

## 2 Significance of the study

This study's significance lies in examining the relationship between renewable energy, CO_2_ emissions, and economic growth and their effect on healthcare expenditure in Asian countries. The study results provide valuable insights for policymakers and public health officials in developing strategies and policies that can help control healthcare expenditure. The study also highlights the importance of considering the regional context in case of development policies and strategies, as the relationship between these factors and healthcare expenditure may vary across different regions in Asia. Additionally, the study contributes to the existing literature on the relationship between environmental factors and healthcare expenditure. The results of the study provide evidence of the negative impact of CO_2_ emissions on healthcare expenditure and the positive impact of renewable energy on healthcare expenditure.

The study can be used to inform policies and strategies aimed at reducing CO_2_ emissions and promoting the use of renewable energy sources. Furthermore, this study can also be used to inform decision-making by healthcare providers, health insurance companies and governments. Healthcare providers can consider the results of the study to develop strategies to reduce healthcare expenditure, while health insurance companies can use the results to inform pricing strategies. Governments can use the results to inform policies and strategies aimed at reducing healthcare expenditure.

## 3 Review of literature

### 3.1 Carbon emissions and health expenditure

The relationship between carbon emissions and health expenditure has gained significant attention in both prosperous and emerging economies following by the literature in the last few decades as the health issues and healthcare costs are rising abruptly. As a result, it is critical to identify the root causes of health problems that affect individuals in order to develop preventive strategies that will preserve the advancement of humanity. The use of energy derived from fossil fuels and CO_2_ emissions are major contributors to health issues and rises in health care costs. Only a few investigations have used statistical methods to examine the relationship between CO_2_ emissions, energy use, health expenditures, and economic growth, despite a growing curiosity in this subject matter among researchers worldwide, particularly in Asian countries that are grappling with how to reduce harmful emissions while also enhancing public wellness.

The first front explores the relationship between carbon emissions and health expenditure. Zaman and Abd-el Moemen [22] state that health expenditure accords more emissions. Yazdi & Khanalizadeh [23] analyzed that carbon monoxide and sulphur oxide have positive effects on human expenditure between the period 1967–2010 in Iran. The recent work by Zaidi & Saidi [24] explained various outcomes, specifically environmental issues mainly carbon emissions pose a serious threat to human health. Further, the study of Boachie et al. [25] also emphasized a positive relationship between GDP and health whereas, a negative relationship between carbon emissions and health expenditure during the period 1970–2008, using the FMOLS technique. The latest study by Li et al. [26] in China describes the rise in pollution-intensive industrial agglomeration enterprises with a sharp rise in urban health expenditure but a steady reduction in rural human expenditure. Jerrett et al. [27] revealed that countries with rapidly increasing emissions rates have high health expenditure costs compared to those with high spending costs on environmental safeguarding practices, eventually resulting in curtailment of health expenditure. Several panel studies established the positive and causal link between health and carbon emissions ([[28], [29], [30]]; Chaabouni, Saidi, and ben Mbarek 2016).

A summary of the literature on carbon emissions and health expenditure is presented in Table 1.

**Table 1:** An overview of the literature on carbon emissions and health expenditure.

Author	Methodology	Country	Time	Key Findings
***Studies show a positive relationship between carbon emissions and health expenditure***.				
Yazdi and Nikos (2014)	ARDL	Iran	1967–2010	Economic growth and all types of emissions show positive relation with health expenditure.
Narayan et al. [28]	Panel Co-integration	8 OECD Countries	1980–1999	Income and carbon monoxide emissions positively impact health expenditure; in the long run, sulphur oxide and emissions positively affect human expenditure.
S. K. Yazdi & Khanalizadeh [23]	ARDL	The Middle East and North African Region (MENA)	1995–2014	Income and all types of Economic Growth and emissions observe a positive effect on human health expenditure.
***Studies show a negative relationship between carbon emission and health expenditure.***				
Lu et al. [31]	Simultaneous Equation Model (SEM)	30 Chinese Provinces	2002–2014	The study shows a negative effect of carbon emissions on public health.
Zaidi & Saidi [24]	ARDL, VEC, VECM Granger Causality Test	Sub-Saharan African Countries	1990–2015	Carbon emissions and Nitrogen oxide emissions negatively harmed human health expenditure.
***Studies show unidirectional and bi-directional relations between carbon emission and health expenditure.***				
Chaabouni et al. [32]	Simultaneous Equation Model (SEM)	51 Countries	1995–2013	There is an uni-directional relationship from carbon emissions to health expenditure except for low-income economies.
Apergis et al. (2018)	Granger Causality	42 Sub-Saharan African Countries	1995–2011	In the long run, health expenditure and carbon emissions depict a bi-directional effect.

### 3.2 Health expenditure and economic growth

The role of energy in developed and developing economies is inevitable and cannot be ignored. The present focus of the study is the relationship between carbon Health expenditure and economic growth. The role of health in developing and developed economies cannot be ignored, as health is a very important factor for any country's economic growth. According to one school of thinking, if a nation develops a solid medical system, its people's welfare and average life span will certainly rise [33,34]. The second strand explores the linkage between economic growth and healthcare expenditure. Atilgan et al. [35], in the context of the Turkish economy for 1975–2013 using the ARDL model, indicated a 1 % increase in healthcare expenses leading to a 0.434 % rise in the country's economic growth. However, a positive relation was found between health expenditure and economic growth in a long duration. Mukherjee [36], using VECM in India, explains the bi-directional co-integration between economic development and healthcare expenditure. It also explores economic expansion costs and healthcare costs are interrelated. Ercelik [37], in the context of the Turkish economy again using ARDL from 1980 to 2015, witnessed co-integration between economic growth and health expenditure in the long run. The heavily increasing GHG, especially carbon emissions, are the result of man-made activities, such as the burning of fossil fuels, causing global warming and more ecological issues. It is quite evident from the previous literature that there is a consequential link between economic growth and carbon emissions which leads to environmental degradation [38,39]. Exploration Studies by Lotfalipour et al. [40] and Ahmad and Du [41] elaborate on the high intensity of carbon emissions due to burning down fossil fuels and increased economic growth and further describe the positive impact of carbon emissions on energy growth.

Table 2 summarises the literature on health expenditures and economic growth.

**Table 2:** An overview of the literature on health expenditure and economic growth.

Author	Methodology	Country	Time	Key Findings
Murthy & Okunade [42]	ARDL	America	1960–2012	The study explains the bi-directional positive co-integration between income, population, and health care. It emphasized medical technology as an important factor in the future.
Piabuo & Tieguhong [43]	Panel OLS Fully Modified OLS Dynamic OLS	African Countries	1995–2015	It also shows the bi-directional relationship between economic growth and healthcare expenditure, revealing that increased income increases healthcare expenditure in the long run.
Dinçer & Yüksel (2019)	Pedroni Panel Cointegration Method and Dumitrescu Hurlin Panel Causality analysis.	European 7 Countries	1996–2016	In the long run, there is no association between healthcare expenditure and economic growth. It also sustains that economic growth is the main reason for healthcare expenditure.
Carrin & Politi [44]	Regression	Least Developed Countries	1985–1990	The findings suggest that a key measure of health spending is the rate of economic growth.
Leidl [45]	Granger Causality Test	European Countries	1974–1994	It observes that economic expansion and healthcare spending are highly associated.
Wang [46]	VECM Quantile Regression Analysis.	31 Different Countries	1986–2007	The study proposes the positive influence of healthcare spending on the economic development of nations. The growth of healthcare expenditure accelerates economic growth.
[2])	Structural Panel VAR Model	22 Different Countries	2004–2013	During dynamic circumstances, health crises at high-income drives Economic development at lower levels can cause economic expansion to halt and lower healthcare costs.
Long et al. [47]	Granger Causality Analysis	China	1952–2012	The research reveals that fossil fuel has a controlling impact on economic growth and carbon emissions.
Mehmood et al.[48]	Pooled Mean Group (PMG) DOLS FMOLS	26 Asian Countries	1990–2012	The result was evident that unitary co-integration between economic growth and healthcare expenditure.
Mardani et al. [49]	Qualitative Systematic Review and Meta-Analysis	Multi-countries	1995–2017	The comprehensive study describes bi-directional causality where economic growth increases/decreases. A substantial reduction in emissions also negatively influences economic growth.

### 3.3 Energy consumption and health expenditure

Since “Health Asset” is a crucial component of human capital, spending on health has taken on a significant amount of significance in the crucial area of public expenditure and public policy. The amount invested in health care may fluctuate from one country to another depending on the economy, sustainability considerations, and other situational variables. The third front explores the relationship between health expenditure and energy consumption.

M. K. Khan et al. [50] explained the impact of energy consumption and health expenditure along with other factors from 1971 to 2016 using ARDL estimates showing the positive correlation between the variables. In the same context Alola, Bekun, and Sarkodie [51], also followed PMG-ARDL analysis from 1997 to 2014 in 16 EU different countries suggesting renewable energy consumption is required to boost a sustainable environment. Hence, it will also enhance medical expenditure as well. The health aspect was also missing from the study by Heidari et al. [52]. They did not offer any information regarding how these factors affected the cost of healthcare in any ASEAN nation, despite examining the relationships between environmental pollution, economic development, and energy use in ASEAN countries [53]. also finds a two-way association between health expenditure and energy consumption by employing the ARDL approach and Granger Causality test in the case of Tunisia during 1990–2011. Apergis & Payne [54] reveal the positive bi-directional relationship between energy consumption and health expenditure in the long run but a substantial impact showing uni-directional in the short-run in France from 1971 to 2004.

Other studies discovered mixed outcomes of the relationship between energy consumption and carbon emissions. Azam [55] analyzed carbon emissions and other variables such as energy use, trade, and human capital in highly intensive carbon emission countries (China, USA, India, and Japan), employing the FMOLS method to state the negative impact on energy consumption during 1971–2013. A bi-directional causality exists between energy consumption and carbon emission following Space State Model and Time-Varying Co-integration Approach in Russia [56]. Ito [57] developed the opposite link, as energy consumption negatively impacts carbon emissions in the short term but positioned the long term in his study, using GMM and PMG models from 2002 to 2011. Table 3 summarises the literature on health expenditures and energy consumption.

**Table 3:** An Overview of the Literature on Energy Consumption and Health expenditure.

Author	Methodology	Country	Time	Key Findings
Qureshi et al. (2015)	2- stage Least square Regression	Malaysia	1975–2012	The research paper indicates that pollutants and other ecological indicators are important in healthcare spending.
Li et al. [58]	Granger Causality Test	China	1965–2015	Bi-directional causations from economic growth to carbon emissions while unidirectional causations examined from economic growth and oil use to carbon emissions.
Qureshi et al. [59]	Panel Cointegration Analysis and OLS	5 Asian Countries	2000–2013	The study explains the positive impact of rising health expenditure in the selected countries. Further suggests that environmental factors accelerate health expenditure.
Ozcan & Ozturk [60]	Bootstrap Panel Causality Test	17 Emerging Countries	1990–2016	Neutral causations from energy consumption to energy growth.
Katrakilidis et al. [61]	VAR	Greece	1960–2012	With continued tailspin expansion and attempts to keep pace with the developed countries, the anticipated decline in healthcare in the coming years may result in increased ecological harm.
[62])	ARDL	EU Countries	1990–2009	Energy Consumption positively impacts energy growth for some countries, though other countries have neutral causality.
Zou et al. [63]	VECM	Low Income Countries	1975–2013	The findings demonstrate that environmental factors and particulate emissions pose a grave threat to the health and prosperity of low-income economies.
Ocal & Aslan [62]	ARDL	Turkey	1990–2010	Energy consumption negatively impacts economic growth. Using the Toda Yamamoto causality Test, an uni-directional causality flows from energy growth to energy consumption.
Lean & Smyth [64]	Panel DOLS Granger Causality Test	ASEAN Countries	1980–2006	The research presented evidence supporting their stance on how energy use influences CO2 emissions, which eventually contributed to a slew of health issues.
Destek & Aslan [65]	Bootstrap Panel Causality	17 Emerging Countries	1980–2012	Mixed causations were found for a different set of countries.
[66]	Cointegration Techniques and Bootstrap Panel unit root tests	MENA Region	1981–2005	Investigated the connection between economic development, environmental degradation, and energy usage. He stated that energy usage was strongly connected to environmental pollution since the use of coal and oil and other mineral wealth harmed the environment and results in a range of health issues in the vicinity.

## 4 Data and research methods

The paper uses the data from 2000 to 2019 for the study. The time period is selected purely based on the availability of the data. Health expenditure, CO_2_ emissions, and GDP per capita and renewable energy consumption variables are used in the study. These variables are extracted from Word Development Indicators (WDI), and World Bank. For more clarity, we reported the variables and their sources in Table 4. The Theoretical foundation for the empirical analysis of the study is IPAT Model. This model was created in 1972 by Paul Ehrlich and John Holdren to explain the relationship between population size (P), wealth (A), and technology (T) and impact (I), or environmental change.

**Table 4:** Description of variables.

Variable	Description	Data Source	
LnHE	Current Health Expenditure per capita in natural logarithm	World Development Indicators	
LnCO2	CO_2_ emission (metric tons per capita) in natural logarithm	World Development Indicators	
LnGDP	GDP per capita (constant 2015 US$) in natural logarithm	World Development Indicators	
LnRE	Renewable energy consumption (% of total final energy consumption) in natural logarithm	World Development Indicators	

Number of countries used and the sub-groupings for the study.

**Table:** 

CENTRAL ASIA	EAST ASIA	WEST ASIA	SOUTH ASIA	SOUTH- EAST ASIA
**Armenia**	China	Cyprus	Bangladesh	Brunei Darussalam
**Kazakhstan**	Korea, Rep.	Jordan	India	Malaysia
**Tajikistan**	Japan	Saudi Arabia	Pakistan	Philippines
**Uzbekistan**	Mongolia	Iraq	Bhutan	Vietnam
**Azerbaijan**		Lebanon	Nepal	Cambodia
**Kyrgyz Republic**		Turkiye	Sri Lanka	Lao PDR
**Turkmenistan**		Israel		Singapore
		Oman		Indonesia
		United Arab Emirates		Myanmar
				Thailand

Rationale Behind the Subgroupings:

This study has utilized the panel data of 36 Asian countries and is further divided into sub-groups for better comparison and clarity. The segregation is based on characteristics such as geographical, demographic, economic, diversity in socio-culture and natural resources. To make the research more relevant, this study proposes a clear distinction of comparison between the economies.

The mathematical model can be written as

(1) $${HEXP}_{\;it}=f\left({CO}_{2\;it},{GDP}_{it},{RE}_{it}\right)$$

where t is the time period from 2000 to 2019, HEXP represents health expenditure per capita, CO_2_ denotes carbon dioxide emission per capita, GDP indicates economic growth, RE is the renewable energy.

Now the econometric model can be represented as

(2) $${HEXP}_{\;it}=\alpha_{0}+\alpha_{1i}{CO}_{2\;it}+\alpha_{2i}{GDP}_{it}+\alpha_{3i}{RE}_{it}+{\;\mu }_{it}$$

(3) $${HEXP}_{\;it}=\alpha_{0}+\alpha_{1i}{CO}_{2\;it}+\alpha_{2i}{lnGDP}_{it}+\alpha_{3i}{lnRE}_{it}+{\;\mu }_{it}$$

In equation (2) represents econometric model and equation (3) denotes that the variables are transformed into natural logarithmic from as per guidance of Anwar et al [67], t represents time period and i denotes the cross – section (1 … 2 … N) and μ is the error term. The coefficients

$\alpha_{1}$, $\alpha_{2}$, and $\alpha_{3}$ characterizes the long run relationship between variables

### 4.1 Methodology

#### 4.1.1 Panel unit root test

The Fisher – ADF, Fisher – PP and IPS panel test statistics were applied to check the unit root due to their robust ability to handle panel data [68,69] and both large and individual datasets [70]. Subsequently, these methods provides a simple multiple – choice option and were utilized to verify for the presence of unit root. These methods are well-defined by the p-values of the individual unit root test for each cross – section and panel [71]. The techniques are expressed in equations (4), (5), (6) as follows:

(4) $$\Delta Z_{it}=\gamma_{0}+\gamma Z_{it}+\sum_{k=1}^{p_{i}}\alpha_{i}\Delta Z_{it-1}+X_{it}\theta +\varepsilon_{it}$$

where $\varepsilon_{it}$ is the residual, i=1,2…N is the cross – section unit that studies the times t=1,2,…, $X_{it}$ denotes the explanatory variables and $p_{i}$ indicates the autoregressive (AR) coefficients and $\varepsilon_{it}$ denotes the random terms. Furthermore, both the ADF and PP are derived from the formula γ=p−1, these statistical tests permit the different order of lag to vary across the section [71]. Finally, the ADF and PP test both allow the conducting of cross – sectional dependence [72].

Additionally, equation (5) indicates both the unit root test for every cross – section and the Fisher – PP test, but it is also essential in robustly evaluating the serial correlation.

(5) $$\varphi =-2\sum_{j=1}^{n}\ln\left(\pi_{i}\right)\to Q^{2}$$

where, $\pi_{i}$ indicates the probability limit from the individual unit root test for cross – section i. The null hypothesis $H_{0}$ of unit root test in the question are not stationary, whereas alternative hypothesis $H_{1}$ denotes that the series is stationary.

(6) $$\Delta {ZA}_{it}=\delta_{i}+\delta_{i}U_{it-1}+\delta_{i}{\bar{XA}}_{t}+\sum_{i=1}^{\sigma }\delta_{i}\Delta {\bar{XA}}_{t-1}+\sum_{i=1}^{\sigma }\delta_{i}\Delta {ZA}_{it-1}+\varepsilon_{it}$$

The IPS unit root test denotes in equation (7), and it is efficient method for representing the problem of heterogeneity and cross – sectional dependence. Furthermore, the study assumes that data series have no serial correlation without specifying the lags.

(7) $$IPS=\frac{1}{N}\sum_{j=1}^{n}{DF}_{i}$$

where ${\bar{XA}}_{t}$ and ${\bar{XA}}_{t-1}$ indicated the cross – section averages, as a result of the selected specified lags. DF denotes the cross – sectional ADF regressions. Moreover, in the case of serial correlation, the IPS suggests applying the ADF t test for individual data series [73].

### 4.2 Cross – sectional dependence test

The root for applying the CD statistical test is that the selected countries are interrelated in various ways such as bilateral trade, economic, geographic (borders sharing) as well as political and socio-cultural contexts [74]. Specified this pretext, several dependencies arise between the countries. Therefore, the Pesaran – CD test, suggested by Pesaran [75] was applied and then applied Breusch-Pegan LM and Pesaran-scaled LM tests, primarily developed by Breusch and Pagan [76]. The cross – sectional dependence test can be represented as:

(8) $$CD=\sqrt{\frac{2T}{N\left(N-1\right)}\;\left(\sum_{i=1}^{N-1}\;\sum_{j=i+1}^{N}{\overset{`}{P}}_{ij}\right)}\;\Rightarrow N\left(0,1\right)$$

where, N and T postulate the cross – sectional magnitude, ${\overset{`}{P}}_{ij}$ denotes the pairwise correlation of the cross – sectional residuals from the ADF test.

### 4.3 FMOLS and DOLS estimates

Finally, we applied FMOS and DOLS technique and analysis the long – run coefficients between the variables in order to check for the robustness of the long – run coefficients. The FMOLS methods take into account the endogeneity problem. The results are robust to endogenous regressors. It also reduces the biasness in the long – run. However, the DOLS techniques was applicable even when the sample size is small and dose separate the simultaneity problem. Furthermore, the obtained cointegrating coefficients through DOLS are asymptotically efficient.

### 4.4 CS-ARDL model

Pesaran's (2015) cross-sectionally augmented autoregressive distributed lag model (CS-ARDL) is used to evaluate the long-run and short-run coefficient relationships. The CS-ARDL approach produces solid findings regardless of the order of integration, whether I (1) or I (0). The CS-ARDL model is an ARDL variant of the dynamic common correlated estimator that accepts individual variables, lagged dependent variables, and cross-section averages while accounting for cross-sectional dependence.

The CS-ARDL relies on the following regression

(9) $$y_{it}=\alpha_{i}+\sum_{l=1}^{p_{y}}\lambda_{l,i}y_{i.t-l}+\sum_{l=0}^{p_{x}}\beta_{l,i}x_{i,t-l}+\sum_{l=0}^{p_{\varphi }}\tau_{l,i}{\bar{z}}_{i,t-l}+\varepsilon_{it}$$

${\bar{z}}_{i,t-l}$ shows lagged cross-sectional averages $\left[{\bar{z}}_{t-l}=\left({\bar{y}}_{i,t-l},{\bar{x}}_{i,t-l}\right)\right]\text{.}$ The mean group coefficient can be written as:

(10) θˆCS−ARDL,i=∑l=0pxβˆl,i1−∑l=0pyλˆl,i,θˆMG=1N∑i=1Nθˆi

where ${\hat{\theta }}_{i}$ describes the individual valuation of the cross-section. Similarly, Error Correction (EC) Equation of the CS-ARDL model can be presented as:

(11) $${\Delta y}_{it}=\sigma_{i}\left[y_{i,t-l}-{\hat{\theta }}_{i}x_{i,t}\right]-\alpha_{i}+\sum_{l=1}^{p_{y}}\lambda_{l,i}y_{i.t-l}+\sum_{l=0}^{p_{x}}\beta_{l,i}x_{i,t-l}+\sum_{l=0}^{p_{\varphi }}\tau_{l,i}{\bar{z}}_{i,t-l}+\mu_{it}$$

In the above equation (11), the EC term indicates the speed of adjustment which is denoted by $\sigma_{i}$.

## 5 Econometrics results

The present section provides econometrics results and discussion. To achieve desired results, step-by-step tests such as descriptive statistics and four different panel unit root tests were applied to check the stationarity of data from different regions. The correlation matrix and FMOLS and DOLS are applied to ascertain the existence of long-run traits in each variable. The two techniques (FMOLS and DOLS) are designed by Philip and Hansen (1990) to dispense optimal estimates and long-term relationships. The descriptive statistics are pursued by a correlational matrix, panel unit root test, cross-section dependence test, cointegration test, and FMOLS and DOLS.

Table 5 presents descriptive analysis in five Asian sub-regions using a panel data from 2000 to 2019. The results show that GDP has a lower mean value compared to other variables, while CO_2_ emissions have the lowest mean value. Renewable energy is negatively skewed across the entire panel. Table 6 examines the association of these variables, and the correlation matrix shows a weak association between health expenditure and renewable energy, but a strong association between health expenditure and carbon dioxide. This suggests that an increase in health expenditure may result in an increase in carbon emissions. The stationarity of the data is evaluated using four different panel unit root tests: Fisher ADF (developed by Ref. [73]), Fisher PP (also developed by Ref. [73]), IPS (developed by Ref. [77]), and LLC (developed by Ref. [78]). These tests were chosen as they have been widely used in previous studies and have been found to be effective in identifying stationarity [[79], [80], [81]].

**Table 5:** Descriptive statistics.

Characteristics	Whole Panel				West Asia			
	LNHE	LNCO2	LNGDP	LNRE	LNHE	LNCO2	LNGDP	LNRE
Mean	1.438	−0.501	24.908	1.829	1.576	−0.586	25.306	0.218
Median	1.404	−0.533	24.915	2.108	1.622	−0.582	25.371	1.022
Maximum	2.383	1.417	30.263	4.538	2.383	0.178	27.588	2.897
Minimum	0.000	−1.984	19.866	−4.605	0.000	−1.684	22.859	−4.605
Std. Dev.	0.422	0.673	2.090	2.282	0.463	0.401	1.335	2.196
Skewness	0.089	0.407	0.130	−1.006	−0.707	−0.439	0.019	−1.016
Kurtosis	2.701	3.027	2.556	3.572	3.553	2.620	1.749	2.957
Jarque-Bera	3.452	18.933	7.541	124.627	16.437	6.532	11.168	29.437
Probability	0.178	0.000	0.023	0.000	0.000	0.038	0.004	0.000
Observations	684	684	684	684	171	171	171	171
	Central Asia				South Asia			
Mean	1.621	0.190	23.392	1.198	1.223	−0.851	24.625	4.044
Median	1.651	0.270	23.132	1.044	1.272	−1.033	24.981	3.966
Maximum	2.359	1.417	26.190	4.168	1.699	0.161	28.625	4.538
Minimum	0.662	−0.914	20.573	−2.996	0.725	−1.844	19.866	3.291
Std. Dev.	0.403	0.655	1.354	2.081	0.225	0.552	2.252	0.354
Skewness	−0.308	0.136	0.169	−0.469	−0.259	0.344	−0.287	−0.033
Kurtosis	2.451	1.912	2.218	2.477	2.197	2.022	2.403	1.820
Jarque-Bera	3.772	6.963	4.018	6.385	4.341	6.793	3.259	6.633
Probability	0.152	0.031	0.134	0.041	0.114	0.033	0.196	0.036
Observations	133	133	133	133	114	114	114	114
	East Asia				South East Asia			
Mean	1.694	−0.338	27.100	1.517	1.213	−0.765	24.905	2.517
Median	1.557	−0.415	28.031	1.497	1.190	−0.661	25.312	3.443
Maximum	2.375	0.847	30.263	3.389	2.026	0.117	27.672	4.451
Minimum	1.256	−1.421	20.852	−0.371	0.616	−1.984	21.272	−4.605
Std. Dev.	0.338	0.757	2.912	0.945	0.340	0.534	1.663	2.219
Skewness	0.745	0.083	−1.004	−0.156	0.416	−0.464	−0.382	−1.565
Kurtosis	2.312	1.494	2.450	2.600	2.762	2.275	1.941	4.980
Jarque-Bera	8.535	7.270	13.719	0.814	5.922	10.992	13.488	108.568
Probability	0.014	0.026	0.001	0.666	0.052	0.004	0.001	0.000
Observations	76	76	76	76	190	190	190	190

**Table 6:** Correlation matrix.

Characteristics	Whole Panel			West Asia				
	LNHE	LNCO2	LNGDP	LNRE	LNHE	LNCO2	LNGDP	LNRE
LNHE	1				1			
LNCO2	0.005	1			−0.415	1		
t-Statistic	0.136	–			−5.923	–		
Probability	0.892	–			0.000	–		
LNGDP	−0.038	−0.091	1		−0.275	−0.086	1	
t-Statistic	−0.989	−2.399	–		−3.719	−1.120	–	
Probability	0.323	0.017	–		0.000	0.265	–	
LNRE	0.001	−0.312	−0.171	1	0.468	−0.472	−0.326	1
t-Statistic	0.029	−8.561	−4.534	–	6.876	−6.964	−4.489	–
Probability	0.977	0.000	0.000	–	0.000	0.000	0.000	–
**Central Asia**				**South Asia**				
LNHE	1.000				1			
LNCO	−0.056	1.000			−0.133	1		
t-Statistic	−0.642	–			−1.420	–		
Probability	0.522	–			0.158	–		
LNGDP	−0.474	0.124	1.000		−0.387	0.554	1	
t-Statistic	−6.168	1.428	–		−4.439	7.049	–	
Probability	0.000	0.156	–		0.000	0.000	–	
LNRE	0.105	−0.685	−0.499	1	0.566	−0.541	−0.900	1
t-Statistic	1.207	−10.765	−6.595	–	7.268	−6.812	−21.848	–
Probability	0.230	0.000	0.000	–	0.000	0.000	0.000	–
**East Asia**				**South East Asia**				
LNHE	1				1.000			
LNCO	−0.863	1.000			−0.099	1.000		
t-Statistic	−14.679	–			−1.360	–		
Probability	0.000	–			0.176	–		
LNGDP	0.554	−0.726	1.000		−0.007	0.321	1.000	
t-Statistic	5.721	−9.094	–		−0.100	4.648	–	
Probability	0.000	0.000	–		0.920	0.000	–	
LNRE	−0.083	0.344	0.172	1.000	0.340	0.100	−0.082	1
t-Statistic	−0.718	3.149	1.503	–	4.962	1.373	−1.127	–
Probability	0.475	0.002	0.137	–	0.000	0.172	0.261	–

The results of the panel unit root tests are reported in Table 7, Table 8 (with intercept and with intercept and trend); by applying all four methods (IPS, ADF, PP, and LLC tests*), it is evident that all variables across different Asian regions have a unit root, indicating that the data is not stationary*. However, after taking the first difference, the variables become stationary and no longer have a unit root. This is evidenced by the *P*-value being less than 0.5 % for almost all the variables across Asian sub-region countries. To further analyze the cross-sectional dependence (CSD) among the variables, the Lagrange multiplier approach given by Breusch and Pagan [76] was applied to all Asian Sub-Region Countries from 2000 to 2019. Table 9 shows that the null hypothesis of no CSD is rejected as there is strong evidence of cross-sectional dependence in all Asian sub-region countries at a 1 % level of significance. The presence of a 1 % significance level indicates a strong cross-sectional dependence between variables; thus, the second-generation panel data method was used for further analysis (see Table 10).

**Table 7:** First-generation panel unit root tests (with intercept).

Variable	Whole Panel							
	Fisher -ADF		Fisher - PP		IPS		LLC	
	I (0)	I (1)	I (0)	I (1)	I (0)	I (1)	I (0)	I (1)
LnHE	72.04	232.53***	76.92	522.27***	−0.07	−9.96***	−1.86**	−9.41***
LnCO2	56.13	201.27***	66.91	359.79***	2.16	−8.36***	−1.75	−8.15***
LnGDP	78.12	147.37***	60.24	237.25***	−0.90	−5.77***	−7.27***	−6.80***
LnRE	39.36	166.55***	38.75	332.66***	5.17	−6.94***	1.92	−4.85***
	**West Asia**							
LnHE	18.03	50.94***	19.93	87.89***	0.47	−4.40**	−0.51	−5.00**
LnCO2	12.96	56.72***	21.36	97.40***	0.97	−4.88***	−0.06	−4.65***
LnGDP	24.19	34.29***	9.34	65.40***	−0.80	−2.58*	−3.88***	−2.17**
LnRE	7.71	36.87***	6.60	68.73***	1.85	−3.36**	0.89	−3.17**
	**Central Asia**							
LnHE	14.75	49.20***	16.19	100.31***	−0.44	−4.73***	−1.22	−5.23***
LnCO2	17.76	51.11***	17.26	82.99***	0.13	−4.93***	−0.86	−6.21***
LnGDP	19.31	19.36***	24.05	28.50***	−1.51	−5.41**	−4.65***	−2.84***
LnRE	18.75	52.64***	16.18	96.38***	−0.71	−5.21***	−1.41*	−5.51***
	**South Asia**							
LnHE	15.428	39.933***	15.517	80.437***	−0.970	−4.242***	−2.131**	−4.241***
LnCO2	5.381	24.263***	11.804	60.221***	1.140	−2.414***	0.508	−1.831***
LnGDP	7.313	38.255***	14.649	52.620***	1.565	−4.004***	−1.766	−5.117***
LnRE	3.215	43.865***	1.202	447.67***	4.271	−4.718***	1.667	−4.049***
	**East Asia**							
LnHE	6.715	15.210**	7.066	50.727***	1.065	−1.788**	0.827	1.105***
LnCO2	4.768	22.929***	3.323	27.135***	1.213	−2.821***	−0.440	−2.427***
LnGDP	8.761	18.321***	5.544	24.916***	−0.650	−2.247***	−2.095	−2.590***
LnRE	6.236	24.128***	4.686	33.508***	1.324	−2.776***	−1.329	−2.815***
	**South East Asia**							
LnHE	17.116	77.245***	18.215	202.909***	−0.136	−6.356***	−2.253**	−6.156***
LnCO2	15.250	46.243***	13.173	92.046***	1.421	−3.457***	−2.051**	−2.990***
LnGDP	18.548	37.144***	6.657	65.814***	−0.494	−2.803***	−4.22***	−3.105***
LnRE	3.445	36.134***	10.078	94.825***	4.439	−2.848***	3.160**	0.449

“***,” “**,” and “*” indicate the level of significance at 1 %, 5 %, and 10 %, respectively.

**Table 8:** First-generation panel unit root tests (with intercept and trend).

Variable	Whole Panel							
	Fisher -ADF		Fisher - PP		IPS		LLC	
	I (0)	I (1)	I (0)	I (1)	I (0)	I (1)	I (0)	I (1)
LnHE	63.35	182.26***	76.95	423.69***	0.23	−7.58***	−1.44**	−7.90***
LnCO2	72.49	156.83***	67.83	339.43***	0.53	−6.09***	−2.27**	−5.93***
LnGDP	25.69	153.55***	16.12	264.44***	4.89	−6.04***	2.51	−9.24***
LnRE	44.09	144.07***	47.55	328.56***	2.67	−5.68***	1.76	−4.91***
	**West Asia**							
LnHE	14.84	43.15***	11.74	94.59***	0.32	−3.62***	−0.71	−4.80***
LnCO2	13.48	43.14***	14.27	98.88***	0.43	−3.47***	−0.22	−3.90***
LnGDP	8.01	34.29***	6.22	64.21***	1.88	−2.49***	1.08	−2.39***
LnRE	11.26	26.05**	9.40	66.16***	0.59	−2.03**	0.61	−2.55**
	**Central Asia**							
LnHE	12.94	36.46***	15.19	78.41***	0.08	−3.43***	−0.61	−4.34***
LnCO2	26.69**	36.76***	29.49**	78.91***	−1.37*	−3.51***	−1.91**	−5.50***
LnGDP	1.64	30.95***	0.67	36.25***	3.37	−2.74***	1.23	−4.64***
LnRE	14.56	44.14***	14.60	94.04***	−0.35	−4.45***	−1.93	−5.04***
	**South Asia**							
LnHE	9.662	29.461***	13.752	74.515***	−0.066	−3.17***	−0.561	−3.16***
LnCO2	3.517	41.876***	6.001	118.763***	1.757	−4.67***	1.157	−3.97***
LnGDP	6.157	39.589***	4.000	77.910***	1.748	−4.32***	0.110	−6.72***
LnRE	2.974	28.265***	1.893	101.412***	2.459	−2.93***	1.558	−2.93***
	**East Asia**							
LnHE	5.328	29.850***	9.835	81.111***	0.517	−4.15***	1.266	2.973***
LnCO2	12.699	17.810**	3.397	20.788***	−1.102	−2.032**	−2.486	−1.398*
LnGDP	6.249	14.512**	2.053	20.250***	0.512	−1.653**	−0.148	−2.213**
LnRE	5.529	25.647***	5.101	40.523***	1.056	−3.44***	−0.025	−5.50***
	**South East Asia**							
LnHE	20.581	62.816***	26.435	139.177***	−0.208	−5.28***	−1.939	−5.72***
LnCO2	16.101	41.117***	14.670	90.364***	1.072	−2.96***	−0.883	−2.08***
LnGDP	3.633	34.206***	3.174	65.821***	3.001	−2.41***	2.672	−4.95***
LnRE	9.770	31.535**	16.558	80.691***	2.156	−2.054**	2.942	3.749**

“***,” “**,” and “*” indicate the level of significance at 1 %, 5 %, and 10 %, respectively.

**Table 9:** Cross-sectional dependence test.

Variable	Whole Panel		Variable	West Asia	
	BP LM	Prob.		BP LM	Prob.
LnHE	3673.28***	0.00	LnHE	197.04***	0.00
LnCO2	4973.87***	0.00	LnCO2	281.67***	0.00
LnGDP	10408.89***	0.00	LnGDP	589.97***	0.00
LnRE	4847.58***	0.00	LnRE	173.31***	0.00
	Central Asia			South Asia	
LnHE	80.31***	0.00	LnHE	80.98***	0.00
LnCO2	141.68***	0.00	LnCO2	59.09***	0.00
LnGDP	368.70***	0.00	LnGDP	271.51***	0.00
LnRE	88.66***	0.00	LnRE	194.31***	0.00
	East Asia			South East Asia	
LnHE	33.65***	0.00	LnHE	286.67***	0.00
LnCO2	70.52***	0.00	LnCO2	422.22***	0.00
LnGDP	66.23***	0.00	LnGDP	795.07***	0.00
LnRE	80.60***	0.00	LnRE	372.05***	0.00

“***,” “**,” and “*” indicate the level of significance at 1 %, 5 %, and 10 %, respectively.

**Table 10:** Second-generation unit root test with intercept and trend.

Variable	Whole Panel				West Asia			
	CADF		CIPS		CADF		CIPS	
	I (0)	I (1)	I (0)	I (1)	I (0)	I (1)	I (0)	I (1)
LnHE	−1.453	−2.497***	−1.769	−4.27***	−1.647	−2.61***	−1.618	−3.38***
LnCO2	−1.484	−2.997***	−1.472	−3.66***	−1.197	−2.54**	−1.464	−3.71***
LnGDP	−2.238***	−2.633***	−2.28***	−3.34***	−2.158	−2.196**	−2.66***	−3.21***
LnRE	−0.925	−2.186***	−0.824	−3.64***	−1.270	−2.62**	−1.428	−3.84***
	**Central Asia**				**South Asia**			
LnHE	−1.100	−2.78***	−1.423	−4.18***	−1.622	−2.397**	−1.552	−4.04***
LnCO2	−1.964	−2.727***	−1.490	−3.87***	−1.24**	−2.69***	−1.824	−4.03***
LnGDP	−2.507**	−2.863***	−2.561**	−3.14***	−1.293	−2.42**	−1.791	−4.28***
LnRE	−1.558	−2.651***	−1.384	−4.65***	−1.102	−2.50**	−1.297	−3.86***
	**East Asia**				**South East Asia**			
LnHE	−0.733	−2.694**	−1.733	−3.85***	−1.247	−2.71***	−1.654	−4.41***
LnCO2	−2.661**	−2.475**	−1.519	−2.39***	−1.130	−2.531**	−1.376	−3.68***
LnGDP	−1.567	−2.534**	−2.415**	−3.669	−2.23***	−2.62***	−2.65***	−3.31***
LnRE	−0.812	−2.91***	−0.744	−3.88***	−0.516	−2.27***	−0.969	−3.82***

“***,” “**,” and “*” indicate the level of significance at 1 %, 5 %, and 10 %, respectively.

Table 11 presents the results of the Pedroni co-integration test, which is used to evaluate the existence of long-term relationships between variables. The test uses seven statistics to validate the null hypothesis of no co-integration. It starts with three non-parametric techniques (Panel V-Static, Panel rho Static, and Panel PP Static) and then a parametric technique (Panel ADF). These four tests assess homogeneity within the dimension, while the other three tests (Group rho static, Group PP Static, and Group ADF) assess heterogeneity between dimensions. The results for the whole panel, as well as for West Asia, Central Asia, South Asia, East Asia, and Southeast Asia, are presented exhaustively. The results show that a majority of the values are larger than the critical value, rejecting the null hypothesis and validating the existence of co-integration among the variables. *It is concluded that Health expenditure, CO_2_, GDP, and Renewable energy are co-integrated in the long term*. The study then uses the Kao co-integration test [82] to check the robustness of the results, and Table 12 presents the results. The t-static values are less than 5 %, providing strong evidence of long-run co-integration between the variables. Further, Johansen Fisher Panel Cointegration test results confirm the presence of long run equlibrium realtionship among variables (see Table 13). Finally, Table 14 describes the Westerlund & Edgerton test [83] and confirms the existence of long-term relationships between the variables. The strong co-integration at the 1 % level among the variables paved the way for further analysis using FMOLS and DOLS (see Table 15, Table 16).

**Table 11:** Pedroni cointegration test.

Whole Panel					
Within-dimension (homogenous)			Between-dimension (heterogeneous)		
Test	Statistics	Prob.	Test	Statistics	Prob.
Panel v-Statistic	−1.112	0.867	Group rho-Statistic	2.139	0.984
Panel rho-Statistic	0.182	0.572	Group *P*P-Statistic	−8.742***	0.000
Panel *P*P-Statistic	−7.245***	0.000	Group ADF-Statistic	−4.057***	0.000
Panel ADF-Statistic	−5.655***	0.000			
	**West Asia**				
Panel v-Statistic	−0.665	0.747	Group rho-Statistic	2.500	0.994
Panel rho-Statistic	1.753	0.960	Group *P*P-Statistic	−4.397***	0.000
Panel *P*P-Statistic	−3.605***	0.000	Group ADF-Statistic	−5.361***	0.000
Panel ADF-Statistic	−5.754***	0.000			
	**Central Asia**				
Panel v-Statistic	−0.951	0.829	Group rho-Statistic	2.049	0.980
Panel rho-Statistic	1.367	0.914	Group *P*P-Statistic	−1.811**	0.035
Panel *P*P-Statistic	−0.699	0.242	Group ADF-Statistic	−2.201**	0.014
Panel ADF-Statistic	−1.588*	0.056			
	**South Asia**				
Panel v-Statistic	−1.038	0.850	Group rho-Statistic	1.480	0.931
Panel rho-Statistic	0.865	0.807	Group *P*P-Statistic	−6.117***	0.000
Panel *P*P-Statistic	−5.413***	0.000	Group ADF-Statistic	−3.856***	0.000
Panel ADF-Statistic	−4.379***	0.000			
	**East Asia**				
Panel v-Statistic	0.272	0.393	Group rho-Statistic	1.714	0.957
Panel rho-Statistic	1.267	0.898	Group *P*P-Statistic	−0.067	0.473
Panel *P*P-Statistic	0.106	0.542	Group ADF-Statistic	−2.029**	0.021
Panel ADF-Statistic	−2.332**	0.010			
	**South East Asia**				
Panel v-Statistic	−1.458	0.928	Group rho-Statistic	0.731	0.768
Panel rho-Statistic	−0.162	0.436	Group *P*P-Statistic	−7.630***	0.000
Panel *P*P-Statistic	−7.623***	0.000	Group ADF-Statistic	−4.338***	0.000
Panel ADF-Statistic	−5.583***	0.000			

“***,” “**,” and “*” indicate the level of significance at 1 %, 5 %, and 10 %, respectively.

**Table 12:** Kao cointegration test.

	Whole Panel		West Asia	
	t-Statistic	Prob.	t-Statistic	Prob.
ADF	−3.768***	0.000	−2.907***	0.002
Residual variance	0.010		0.014	
HAC variance	0.011		0.015	
	**Central Asia**		**South Asia**	
ADF	−1.411**	0.079	−2.176**	0.0148
Residual variance	0.013		0.005	
HAC variance	0.015		0.004	
	**East Asia**		**South East Asia**	
ADF	−2.260**	0.0119	−1.05142	0.1465
Residual variance	0.004		0.009351	
HAC variance	0.004		0.009619	

“***,” “**,” and “*” indicate the level of significance at 1 %, 5 %, and 10 %, respectively.

**Table 13:** Johansen Fisher panel cointegration test.

Test	Whole Panel				West Asia			
	Fisher Statistics		Fisher Statistics		Fisher Statistics		Fisher Statistics	
	(from trace test)	Prob.	(from max-eigen test)	Prob.	(from trace test)	Prob.	(from max-eigen test)	Prob.
None	565.7***	0.000	412.1***	0.000	127.2***	0.00	88.62***	0.00
At most 1	238.9***	0.000	170.9***	0.000	56.7***	0.00	35.38**	0.01
At most 2	132.4***	0.000	103.0**	0.006	37.1**	0.01	27.09*	0.08
At most 3	135.8***	0.000	135.8***	0.000	41.7***	0.00	41.73***	0.00
	Central Asia				South Asia			
None	81.9***	0.000	65.3***	0.000	97.4***	0.000	73.2***	0.000
At most 1	31.9***	0.004	21.6*	0.087	39.3***	0.000	28.6**	0.005
At most 2	22.0*	0.079	16.2	0.297	21.0*	0.050	17.970	0.117
At most 3	29.1**	0.010	29.1**	0.010	18.9*	0.090	18.9*	0.090
	East Asia				South East Asia			
None	107.40***	0.00	68.27***	0.00	178.40***	0.00	135.00***	0.00
At most 1	52.39***	0.00	34.47***	0.00	69.81***	0.00	56.28***	0.00
At most 2	27.68***	0.00	21.95**	0.01	32.72**	0.04	24.99	0.20
At most 3	19.04**	0.01	19.04**	0.01	34.56**	0.02	34.56**	0.02

“***,” “**,” and “*” indicate the level of significance at 1 %, 5 %, and 10 %, respectively.

**Table 14:** Westerlund & Edgerton cointegration test.

Statistic	Whole Panel			West Asia		
	Value	Z	*P*-value	Value	Z	*P*-value
Gt	−2.025**	−1.85	0.032	−1.608	0.296	0.616
Ga	−3.797	3.856	1.00	−2.948	2.336	0.99
Pt	−7.918	−0.012	0.495	−3.784	0.127	0.551
Pa	−3.141	1.072	0.858	−3.706	0.268	0.606
	Central Asia			South Asia		
Gt	−2.048	−0.874	0.191	−2.341**	−1.512	0.065
Ga	−6.257	0.658	0.745	−3.311	1.765	0.961
Pt	−2.789	0.53	0.702	−4.769	−1.178	0.119
Pa	−1.379	1.211	0.887	−3.528	0.288	0.613
	East Asia			South East Asia		
Gt	−0.532	2.299	0.989	−2.779**	−3.303	0.001
Ga	−1.539	2.008	0.978	−4.026	1.916	0.972
Pt	−1.062	1.2	0.885	−7.991**	−2.92	0.002
Pa	−2.095	0.689	0.755	−4.622	−0.176	0.43

“***,” “**,” and “*” indicate the level of significance at 1 %, 5 %, and 10 %, respectively.

**Table 15:** FMOLS model results.

Variables	Coefficient	Std. Error	Prob.
Whole Panel			
LNCO2	0.058***	0.015	0.000
LNGDP	0.032***	0.007	0.000
LNRE	−0.086***	0.012	0.000
West Asia			
LNCO2	0.498***	0.039	0.000
LNGDP	0.047***	0.016	0.005
LNRE	−0.169***	0.028	0.000
Central Asia			
LNCO2	0.192***	0.033	0.000
LNGDP	0.043*	0.025	0.083
LNRE	−0.016	0.045	0.724
South Asia			
LNCO2	0.343*	0.056	0.000
LNGDP	0.008**	0.021	0.703
LNRE	−0.034**	0.036	0.343
East Asia			
LNCO2	0.022	0.069	0.749
LNGDP	0.150***	0.035	0.000
LNRE	−0.369***	0.051	0.000
Southeast Asia			
LNCO2	0.256***	0.026	0.000
LNGDP	0.047***	0.013	0.001
LNRE	−0.014	0.021	0.500

“***,” “**,” and “*” indicate the level of significance at 1 %, 5 %, and 10 %, respectively.

**Table 16:** DOLS model results.

Variables	Coefficient	Std. Error	Prob.
Whole Panel			
LNCO2	0.109***	0.040	0.007
LNGDP	0.066***	0.012	0.000
LNRE	−0.062***	0.020	0.003
West Asia			
LNCO2	0.461***	0.153	0.004
LNGDP	0.091**	0.042	0.035
LNRE	−0.195***	0.054	0.001
Central Asia			
LNCO2	0.296*	0.168	0.085
LNGDP	0.075	0.068	0.275
LNRE	−0.397*	0.191	0.045
South Asia			
LNCO2	0.430***	0.087	0.000
LNGDP	0.011	0.025	0.666
LNRE	−0.149	0.227	0.516
East Asia			
LNCO2	0.095	0.264	0.722
LNGDP	0.143***	0.039	0.002
LNRE	−0.258***	0.035	0.000
Southeast Asia			
LNCO2	0.407***	0.105	0.000
LNGDP	0.123***	0.026	0.000
LNRE	−0.027**	0.015	0.079

“***,” “**,” and “*” indicate the level of significance at 1 %, 5 %, and 10 %, respectively.

The results of the study, analyzed the effects of renewable energy, CO_2_ emissions, and economic growth on healthcare expenditure in Asian countries, are presented in Table 15. *According to the analysis, a 1 % increase in CO2 emissions is associated with a 0.058 % increase in healthcare expenditure that indicating that increasing rate of CO_2_ emissions led to environmental deprivation which further causes an increase in health expenditure*. The higher rate of CO_2_ emissions is directly associated with increased energy production which results environmental pollution and causing population sick that ultimately enhanced health expenditure. The findings of the study is consistent with the studies Yazdi and Nikos (2014), Narayan et al [28], Yazdi and Khanalizadeh [23] Shahzad et al. [84]. The results of the study show that 1 % increase in economic growth is linked to a 0.032 % increase in healthcare expenditure. The findings of the study are in line to the other studies [[42], [43], [49], [85]]. On the other hand*, an increase in renewable energy is found to be associated with a decrease in healthcare expenditure, with a 1 % increase resulting in a -0.086 % decrease*. The empirical findings of this study confirm that health care expenditure reduces as a result of adoption of renewable energy in Asian countries. The findings of this study is similar with various developed and developing economies [[58], [61], [62], [86]].

In West Asian countries, the study found that a 1 % increase in CO2 emissions is associated with a 0.498 % increase in healthcare expenditure, while a 1 % increase in economic growth is associated with a 0.047 % increase in healthcare expenditure. The findings of the study is in tune with [84]. The increase in healthcare expenditure due to economic growth is less than the increase due to CO2 emissions. Additionally, an increase in renewable energy is found to have a negative association with healthcare expenditure, with a 1 % increase resulting in a −0.169 % decrease. The results is consistent with the findings [61].

In Central Asia, the study found that an increase in CO_2_ emissions is associated with a higher healthcare expenditure, specifically a 1 % increase in CO_2_ emissions is found to result in a 0.192 % increase in healthcare expenditure. This finding is in agreement with studies conducted in Europe and Central Asia and Pakistan (Hsu, 2013; [84]). However, the relationship *between economic growth and healthcare expenditure, as well as the relationship between renewable energy and healthcare expenditure, is not statistically significant*.

In South Asia, the study found that an increase in CO_2_ emissions is associated with an increase in healthcare expenditure (Khan, J., & Khattak, 2022), while the relationship between economic growth and healthcare expenditure is not statistically significant. Additionally, an increase in renewable energy is associated with a decrease in healthcare expenditure but not at a very significant level.

In East Asia, the study found that the relationship between CO_2_ emissions and healthcare expenditure is not statistically significant, while an increase in economic growth is associated with an increase in healthcare expenditure and an increase in renewable energy is associated with a decrease in healthcare expenditure.

In Southeast Asia, the results indicate that an increase in CO_2_ emissions is significantly associated with higher healthcare expenditure at the level of 0.1 %. Specifically, a 1 % increase in CO2 emissions is found to result in a 0.256 % increase in healthcare expenditure. This finding is in tune with other studies such as *in Middle East and North Africa region (MENA) by* 23*, in Fiji by* 28*, and in Pakistan by* 84*).* The increase in economic growth is also associated with an increase in healthcare expenditure, with a 1 % increase resulting in a 0.047 % increase, and is significant at the level of 0.1 %. This study is in congruence with earlier studies conducted in US [42], central African states (CEMAC) and selected African countries [43], E7 Countries [85] and developed and developing countries [49]*. However, the coefficient of renewable energy is -0.014, which means that an increase in renewable energy is not associated with a significant change in healthcare expenditure in Southeast Asia, as it is not statistically significant at a level of 5 %. Overall, the results suggest that in Southeast Asia, an increase in CO2 emissions is associated with an increase in healthcare expenditure,* while an increase in economic growth is also associated with an increase in healthcare expenditure and an increase in renewable energy is not associated with significant change in healthcare expenditure.

The findings of the DOLS model, presented in Table 16, support the results of the FMOLS model shown in Table 15. This confirms the robustness of the results obtained from the FMOLS model. In the last part of the study in Table 17, with the help of the CS-ARDL model, this study shows that carbon emissions and GDP have increased healthcare expenditure. In contrast, renewable energy consumption has decreased healthcare expenditure in both the long and short run.

**Table 17:** CS-ARDL test results.

Variables	Coefficient	Standard Error	t-Statistic	Probability Value
Long-run Equation				
CO2	0.149**	0.053	2.811	0.026
GDP	0.294***	0.043	6.83	0.000
RE	−0.165***	0.026	−6.34	0.000
Short-run Equation				
HE lag	0.239**	0.131	1.824	0.049
CO2	0.276**	0.112	2.46	0.031
GDP	0.217***	0.041	5.292	0.000
RE	−0.193***	0.021	−9.194	0.000
ECT	−0.591***	0.131	−4.511	0.000
F Stat.	4.97 (0.00)			
R-squared	0.56			

The results of the DOLS model indicate that the relationship between the independent variables, such as renewable energy, CO_2_ emissions, and economic growth, and the dependent variable, healthcare expenditure, are consistent with the results of the FMOLS and CS-ARDL model. This suggests that the results of the study are not sensitive to the estimation method used and can be considered reliable.

## 6 Conclusion and policy implications

This study explores how economic growth, CO_2_, and renewable energy affect healthcare costs across a panel of 36 Asian nations. Results of the investigation indicate that a higher rate of CO_2_ emissions is closely linked to more energy being produced, which in turn causes pollution in the environment and increases the number of sick people, which in turn raises health care costs. According to the study's findings, healthcare spending in Asian countries is decreasing as a result of the use of renewable energy.

Environmental degradation and exhaustive energy use have changed the future of the planet, facing major natural disasters such as floods, drought, rains, earthquakes, etc. due to global warming and climate change. Not only developing nations but developed nations also suffer from serious natural calamities. Researchers from all over the world trying their best to mitigate the effects of greenhouse gases, particularly carbon dioxide. In the above-mentioned literature, various attempts have been made to minimize harmful carbon emissions impact on human health by energy efficiency though there are certain hurdles on the way out in the long run. The relationship between health spending and economic growth was perceived as being one of luxury, indicating that health care is a product like certain other goods, and government participation in the healthcare industry. The FMOLS characteristics demonstrate that health care spending in the Asian countries lowers CO_2_ emissions. Furthermore, this study found statistical support for the claim that spending on the health sector reduced contamination of the environment in Asian countries.

### 6.1 Policy implications

The findings of this study have several policy implications for policymakers and public health officials in Asian countries. Firstly, the study's results suggest that increasing CO_2_ emissions are associated with an increase in healthcare expenditure in all regions of Asia. This highlights the importance of implementing policies and strategies that aim to reduce CO_2_ emissions in order to control healthcare expenditure. Therefore, the study recommends that in order to achieve equitable economic expansion, policymakers must increase health spending through environmental protection and improvement. Therefore, the government should think about controlling the expense and usage of healthcare facilities, raising the amount of money spent on funds and wellness care, and developing guidelines for better cost-utilization schemes that take into consideration medical facilities, Infrastructures, medical debris, environmentally conscious administration of hospitals, and use of power from sources of clean electricity as it includes promoting the use of renewable energy sources, implementing energy-efficient practices, and implementing policies that encourage sustainable development.

Secondly, the study's findings indicate that the relationship between economic growth and healthcare expenditure varies across different regions in Asia. Clean energy sources should take precedence over fossil fuels in the generation of electricity. Energy derived from renewable sources, such solar power, should be supplied to medical centers in order to reduce carbon emissions over time, heavily influencing economic growth needed for the healthcare sector. As a result, their financial stress and carbon emissions will be eased. In some regions, economic growth is found to be associated with an increase in healthcare expenditure, while in others it is not. This also suggests that policymakers should be careful when developing economic policies and strategies keeping in view the expenditure and consumption of health facilities, as they may have unintended effects on healthcare expenditure.

Lastly, the study found that the relationship between renewable energy and healthcare expenditure also varies across different regions in Asia. In some regions, an increase in renewable energy is associated with a decrease in healthcare expenditure, while in others it is not. Asian countries should encourage the adoption of more stringent commercial rules and regulations that support environmental sustainability and encourage ecologically friendly conduct in larger economies. It is also recommended that corporations that produce renewable energy be offered enticing subsidies. The amount of capital needed to generate renewable energy seems large. Finance for renewable energy must be ensured, and management must be courted to endorse it. It is advised to offer distinctive incentives, such as a tax break, to entice people to transition to clean energy sources. Businesses that use fossil fuels extensively ought to be subject to greater taxes. This highlights the importance of considering the regional context when developing energy policies and strategies.

In holistic sense, this study's results suggest that policymakers and public health officials should consider the regional context when developing policies and strategies to control healthcare expenditure. Furthermore, policies and strategies that aim to reduce CO_2_ emissions and promote renewable energy should be given priority in order to help control healthcare expenditure in Asian countries.

## 7 Limitations and directions for further research

The sample size of the study is small due to data limitations. Future research can yield more accurate parameter estimates by boosting the sample size. The study's research was based on per capita carbon emissions and was restricted to the Asian region, spanning 2000 to 2019. Future researchers may consider expanding the investigation to include consumption- or territory-based CO_2_ emissions as data becomes accessible. The findings of this study may be confirmed or refuted by other research using panel data for the continents of Europe, America, or Africa. Research providing data from different areas and contaminants will be beneficial in influencing the debate about healthcare costs, renewable energy, environmental deterioration, and economic growth.

## Consent to participate

Not applicable.

## Consent to publish

Not applicable.

## Data availability

Data used in this study can be found in the cited link.

## Ethical approval

This study followed all ethical practices during writing.

## Funding

This study received no specific financial support.

## CRediT authorship contribution statement

**Preeti Slathia:** Data curation, Conceptualization, Formal analysis, Methodology. **Ashutosh Vashishtha:** Writing – original draft, Supervision, Conceptualization, Project administration. **Pabitra Kumar Jena:** Project administration, Supervision, Writing – original draft. **Pritish Kumar Sahu:** Writing – review & editing.

## Declaration of competing interest

We hereby affirm that the content of this manuscript are original. Furthermore it has been neither published elsewhere fully or partially or any language nor submitted for publication (fully or partially) elsewhere simultaneously. We affirm that the all author (s) have seen and agreed to the submitted version of the paper and their inclusion of name(s) as co-author(s). Therefore, you are free to publish our Research Paper in your Journal.

The authors declare that they have no known competing financial interests or personal relationships that could have influenced the work reported in this paper. Preeti Slathia contributed to the study conception, design, material preparation, data collection and analysis. Ashutosh Vashishtha commented on final editing and formatting of the manuscript and Pabitra Kumar Jena is responsible for the literature review, conclusion and policy recommendations. Finally, Dr. Pritish Kumar Sahu is responsible for the revision and upgradation of the econometric techniques. All authors read and approved the final manuscript.
